# Inhibition of Morphine- and Ethanol-Mediated Stimulation of Mesolimbic Dopamine Neurons by *Withania somnifera*

**DOI:** 10.3389/fnins.2019.00545

**Published:** 2019-06-04

**Authors:** Valentina Bassareo, Giuseppe Talani, Roberto Frau, Simona Porru, Michela Rosas, Sanjay B. Kasture, Alessandra T. Peana, Eleonora Loi, Enrico Sanna, Elio Acquas

**Affiliations:** ^1^Department of Biomedical Sciences, University of Cagliari, Cagliari, Italy; ^2^Centre of Excellence on Neurobiology of Addiction, University of Cagliari, Cagliari, Italy; ^3^Institute of Neuroscience, National Research Council, Cagliari, Italy; ^4^Department of Life and Environmental Sciences, University of Cagliari, Cagliari, Italy; ^5^Pinnacle Biomedical Research Institute, Bhopal, India; ^6^Department of Chemistry and Pharmacy, University of Sassari, Sassari, Italy

**Keywords:** dopamine, ethanol, morphine, nucleus accumbens shell, standardized *Withania somnifera* extract, ventral tegmental area, GABA

## Abstract

Morphine- and ethanol-induced stimulation of neuronal firing of ventral tegmental area (VTA) dopaminergic neurons and of dopamine (DA) transmission in the shell of the nucleus accumbens (AcbSh) represents a crucial electrophysiological and neurochemical response underlying the ability of these compounds to elicit motivated behaviors and trigger a cascade of plasticity-related biochemical events. Previous studies indicate that the standardized methanolic extract of *Withania somnifera* roots (WSE) prevents morphine- and ethanol-elicited conditioned place preference and oral ethanol self-administration. Aim of the present research was to investigate whether WSE may also interfere with the ability of morphine and ethanol to stimulate VTA dopaminergic neurons and thus AcbSh DA transmission as assessed in male Sprague-Dawley rats by means of patch-clamp recordings in mesencephalic slices and *in vivo* brain microdialysis, respectively. Morphine and ethanol significantly stimulated spontaneous firing rate of VTA neurons and DA transmission in the AcbSh. WSE, at concentrations (200–400 μg/ml) that significantly reduce spontaneous neuronal firing of VTA DA neurons via a GABA_A_- but not GABA_B_-mediated mechanism, suppressed the stimulatory actions of both morphine and ethanol. Moreover, *in vivo* administration of WSE at a dose (75 mg/kg) that fails to affect basal DA transmission, significantly prevented both morphine- and ethanol-elicited increases of DA in the AcbSh. Overall, these results highlight the ability of WSE to interfere with morphine- and ethanol-mediated central effects and suggest a mechanistic interpretation of the efficacy of this extract to prevent the motivational properties of these compounds.

## Introduction

*Withania somnifera* (*WS*) Dunal (family Solanaceae), also known as *Ashwagandha* or *Indian Ginseng*, is a plant widely used in traditional Ayurvedic medicine in India since antiquity (Kulkarni and Dhir, 2008). It is included in the Indian Pharmacopoeia (Singh et al., 2011) as a safe official medication for the treatment of several ailments (Dar et al., 2016) and recent evidence supports its anti-inflammatory, immunomodulatory, neuroprotective (Bhatnagar et al., 1975; Kulkarni et al., 2008; Dar et al., 2016; Yenisetti et al., 2016) and free-radical scavenging (Bhattacharya et al., 2001; Davis and Kuttan, 2001; Prakash et al., 2014; Dar et al., 2015) properties. Moreover, *WS* has been shown to modulate GABAergic [(γ-amino-butyric acid (GABA)] (Mehta et al., 1991; Kulkarni and Dhir, 2008) and cholinergic (Schliebs et al., 1997) neurotransmission and to affect different properties of addictive drugs. In fact, several studies have reported that *WS* prevents the development of tolerance and dependence to morphine (Kulkarni and Ninan, 1997) as well as morphine withdrawal-dependent reduction of dendritic spine density in the shell of the nucleus accumbens (AcbSh) (Diana et al., 2006; Kasture et al., 2009). In addition, *WS* has also been shown to reduce anxiety associated with ethanol-withdrawal and increase ethanol-induced anxiolysis (Gupta and Rana, 2008).

Drug addiction is defined as the progressive loss of control over drug taking and results from a long series of neuroadaptations taking place in selective neural circuits in response to the progressive exposure to addictive drugs (Volkow and Morales, 2015). Notably, among these drugs both morphine (Di Chiara and Imperato, 1988) and ethanol (Imperato and Di Chiara, 1986; Bassareo et al., 2003) preferentially increase dopamine (DA) transmission in the AcbSh, a property that has been pinpointed as a neurochemical feature shared by all substances with addictive potential (Di Chiara, 1999; Di Chiara et al., 2004). Moreover, this property represents the neurophysiological correlate of increased spontaneous firing rate of DA neurons in the ventral tegmental area (VTA) by addictive drugs, including morphine (Jalabert et al., 2011; Chen et al., 2015) and ethanol (Gessa et al., 1985; Brodie et al., 1990; Brodie and Appel, 1998; Xiao and Ye, 2008; Theile et al., 2011) on the one hand, and of morphine- and ethanol-mediated motivated behaviors (Di Chiara, 1999; Di Chiara et al., 2004) on the other. However, in spite of decades of research aimed at understanding its neurobiological basis, drug addiction represents a complex pathological condition for which a still limited list of therapeutic tools, restricted mostly to opioids, nicotine and alcohol dependence, is available (Lyon, 2017). Hence, the preclinical search of new therapeutic approaches, including the application and development of strategies based on the use of substances obtained from herbal remedies or from whole herbal extracts (Carai et al., 2000; Lu et al., 2009; Liu et al., 2011; Zhang et al., 2014), appears nowadays highly desirable. In this regard, the standardized methanolic extract of *WS* roots (*WSE*) has recently been shown to affect the ability of morphine (Ruiu et al., 2013) and ethanol (Spina et al., 2015) to elicit acquisition and expression of conditioned place preference (CPP) in mice as well as the acquisition and maintenance of oral ethanol self-administration in rats (Peana et al., 2014). However, in spite of the behavioral evidence gathered on the efficacy of *WSE* to significantly reduce the affective and motivational impact of the acute administration of morphine and ethanol, no studies have attempted yet to clarify the mechanism(s) by which such effects of *WSE* may take place. In this regard, one of the most likely candidate mechanism by which *WSE* impairs acquisition and expression of morphine- and ethanol-elicited CPP (Ruiu et al., 2013; Spina et al., 2015) and acquisition and maintenance of ethanol oral self-administration (Peana et al., 2014) is represented by the ability of *WSE* to affect morphine- and ethanol-elicited increase of the spontaneous firing rate of VTA DA neurons as well as of phasic DA transmission in the AcbSh.

Hence, in order to shed light on the mechanism(s) by which *WSE* prevents DA-mediated morphine- and ethanol-elicited motivated behaviors (Ruiu et al., 2013; Peana et al., 2014; Spina et al., 2015), the present study aimed at characterizing further the ability of *WSE* to affect the psychopharmacological properties of morphine and ethanol by assessing whether it may also interfere with the ability of these substances to stimulate VTA DA neuronal firing activity and prevent morphine- and ethanol-elicited increases of DA transmission in the AcbSh. In addition, to further characterize the mechanism by which *WSE* may affect the firing activity of VTA DA neurons by itself, we also applied *WSE* in the presence of GABA_A_ and GABA_B_ receptors antagonists, bicuculline and SCH50911, respectively. To this end, *ex vivo* standard patch-clamp recordings of VTA DA neurons in mesencephalic slices and *in vivo* brain microdialysis in the AcbSh, from male Sprague-Dawley rats, were performed.

## Materials and Methods

### Subjects

Male Sprague-Dawley rats (Envigo, Italy) bred in our animal facility (19–30 days of age) and male Sprague-Dawley rats (Envigo, Italy) (45–52 days of age) were used for the electrophysiological and the microdialysis experiments, respectively. Animals had access to water and standard laboratory food (Stefano Morini, S. Polo D’Enza, RE, Italy) *ad libitum*. Animal care and handling throughout the experimental procedures were in accordance with the guidelines for care and use of experimental animals of the European Community Council (2010/63/UE L 276 20/10/2010) and with Italian law (DL 04.03.2014, N° 26). In particular, this study was approved by the Organization for Animal Care of the University of Cagliari (OPBA-UniCA) and performed in accordance with the Ministry of Health authorization number 1177/2016-pr (December 15, 2016). Every effort was made to minimize suffering and reduce the number of animals used.

### Experimental Procedures for Electrophysiological Experiments

#### Preparation of Rat VTA Slices

Brain slices were prepared as previously described by Talani et al. (2011, 2016). In brief, at post-natal day (PND) 19–30, animals were subjected to 5% isoflurane deep anesthesia and decapitated. Brains were rapidly removed from the skull and transferred into a modified artificial cerebrospinal fluid (aCSF) solution containing (in mM): 220 sucrose, 2 KCl, 0.2 CaCl_2_, 6 MgSO_4_, 26 NaHCO_3_, 1.3 NaH_2_PO_4_, and 10 D-glucose (pH 7.4, set by aeration with 95% O_2_ and 5% CO_2_). Horizontal brain slices containing the VTA (thickness, 260 μm) were cut in ice-cold modified aCSF with the use of a Leica VT1200S vibratome (Leica, Heidelberg, Germany). The slices were then transferred immediately to a nylon net submerged in standard aCSF containing (in mM): 126 NaCl, 3 KCl, 2 CaCl_2_, 1 MgCl_2_, 26 NaHCO_3_, 1.25 NaH_2_PO_4_, and 10 D-glucose (pH 7.4, set by aeration with 95% O_2_ and 5% CO_2_). After an incubation for at least 40 min at the controlled temperature of 35°C and a subsequent waiting for at least 1 h at room temperature, hemi-slices were transferred to the recording chamber and continuously perfused with standard aCSF at a constant flow rate of ∼2 ml/min. For all recordings, the temperature of the bath was maintained at 33°C.

#### Patch-Clamp Recordings

Patch-clamp recordings from VTA dopaminergic neurons were performed as previously described by Dazzi et al. (2014). Recording pipettes were prepared from borosilicate capillaries with an internal filament with the use of a P-97 Flaming Brown micropipette puller (Sutter Instruments, Novato, CA, United States). Resistance of the pipettes ranged from 4.5 to 6.0 MΩ when they were filled with the following solution (in mM): 135 potassium gluconate, 10 MgCl_2_, 0.1 CaCl_2_, 1 EGTA, 10 Hepes-KOH (pH 7.3), and 2 ATP (disodium salt). Signals were recorded with the use of an Axopatch 200-B amplifier (Axon Instruments Inc., San Jose, CA, United States), filtered at 2 kHz, and digitized at 5 kHz. The pClamp 9.2 software (Molecular Devices, Union City, CA, United States) was used in order to measure and analyze the firing rate and other membrane kinetic parameters of VTA neurons as well as the occurrence of HCN-mediated *I*_h_ currents (see below). The cell-attached configuration was used for monitoring the spontaneous firing rate in control condition (baseline) as well as during and after drug application. After obtaining a pipette-membrane seal with a GΩ resistance, at least 10 min were allowed prior recording, in order to have a stable and regular baseline firing rate. At the end of each recording, the whole-cell configuration was obtained to determine the presence of *I*_h_ currents, in order to confirm the identity of VTA DA neurons. In fact, as also previously reported by Grace and Onn (1989), in the present experiments DA neurons in the VTA were identified by the presence of both an *I*_h_ current (mean amplitude: -185 ± 43 pA, *n* = 40) evoked in response to a single hyperpolarizing voltage step, from -65 to -115 mV, and a regular firing rate of spontaneous action potentials (4.84 ± 0.4 Hz, *n* = 40).

### Experimental Procedures for Microdialysis Experiments

#### Surgery

Beginning 3 days before surgery, rats were handled once daily for 5 min to habituate them to the contact with the operator and to the experimental procedures (intraperitoneal and gavage administrations). Under deep anesthesia by equitesin, as reported by Bassareo et al. (2015), a vertical microdialysis probe was stereotaxically and unilaterally implanted in the AcbSh, randomly in the left or in the right hemisphere, using the following coordinates: AP: 2.0 mm from bregma, ML: 1 mm from bregma and DV: -7.6 mm from dura, according to the rat brain atlas of Paxinos and Watson (1998). After surgery, rats were housed in individual hemispheric cages under the same standard animal facility conditions, left undisturbed for at least 24 h and fed daily with 20 g of standard chow (their weight being maintained at approximately 95% of their *ad libitum* weight). Water was available *ad libitum* throughout the duration of experiments.

#### Probe Preparation

Vertical dialysis probes were prepared with AN69 fibers (Hospal Dasco, Bologna, Italy), according to the method of Di Chiara et al. (1993) as modified by Tanda et al. (1996). The length of the dialyzing portion of the probe was 1.5 mm.

#### Microdialysis Experiments

On the day of the experiment, probes were connected to an infusion pump and perfused with Ringer’s solution (in mM): 147 NaCl, 4 KCl, 2.2 CaCl_2_ (see Lecca et al., 2006 on the use of 2.2 mM Ca^2+^ in the Ringer) at the constant rate of 1 μl/min. Dialysate samples (10 μl) were taken every 10 min and injected without purification into either a high-performance liquid chromatograph (HPLC) or an ultra HPLC (UHPLC; ALEXYS Neurotransmitter analyzer, Antec, Zoeterwoude, Netherlands). The HPLC was equipped with a reverse phase column (LC-18 DB, 15 cm, 5 μm particle size, Supelco, Waters, Milford, MA, United States) and a coulometric detector (ESA, Coulochem II, Bedford, MA, United States) to quantify DA. The first electrode of the detector was set at +125 mV (oxidation) and the second at -175 mV (reduction). The composition of the mobile phase was (in mM): 50 NaH_2_PO_4_, 0.1 Na_2_-EDTA, 0.5 n-octyl sodium sulfate, 15% (v/v) methanol, pH 5.5 (obtained by adding Na_2_HPO_4_). Under these conditions, sensitivity of the assay for DA was 5 femto-moles (fmol)/sample. The UHPLC was equipped with a NeuroSep (C18 110, 1.0 × 100 mm, 1.7 μm) column (Supelco, Waters, Milford, MA, United States) and an electrochemical amperometric detector (DECADE II SCC) (Antec, Zoeterwoude, Netherlands). Composition of the mobile phase was (in mM): 100 phosphoric acid, 100 citric acid, 0.1 EDTA, 8% (v/v) acetonitrile. Using these conditions sensitivity of the assay for DA was 5 fmol/sample. Every subject was administered, for pre-treatment (saline or *WSE*) and treatment (saline or tap water or morphine or ethanol) only once during the experimental session.

#### Histology

**Figure 1:**
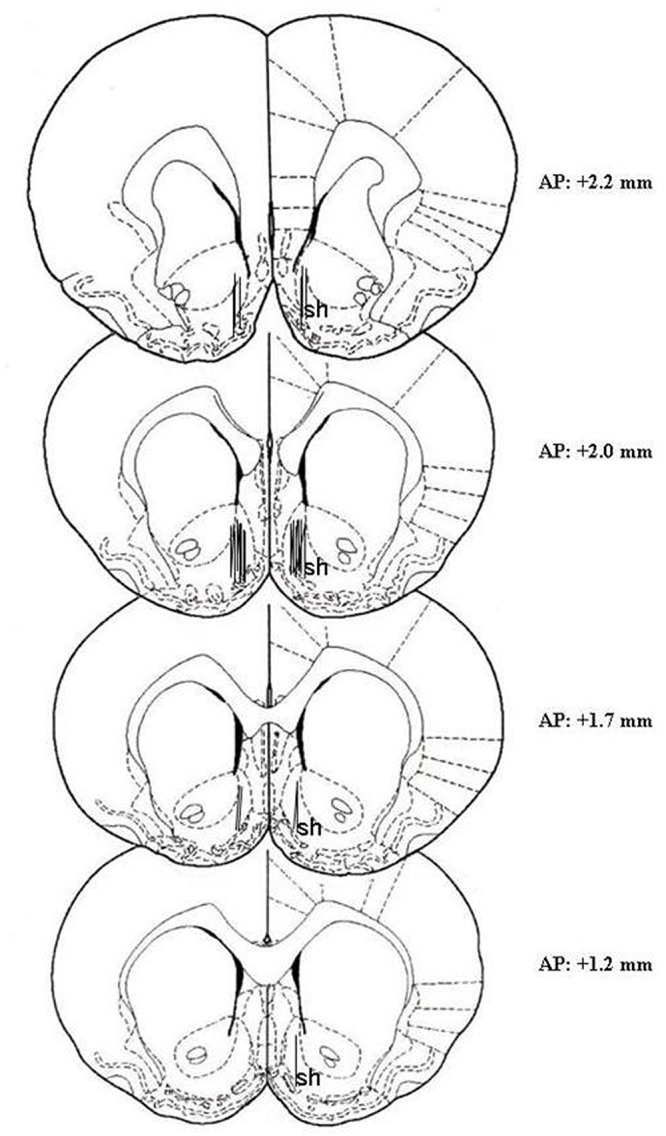
Localization of dialysis probes (dialyzing portion) within the AcbSh. Representative images of the probe location (drawn after histological examination) in the brain atlas plates showing the AcbSh (sh) at different AP distances from bregma according to the rat brain atlas of Paxinos and Watson (1998).

At the end of the microdialysis experiment, rats were anesthetized as reported by Bassareo et al. (2015), probes were removed and the brains were kept in a 4% (w/v) formaldehyde solution for at least 1 week and successively cut with a vibratome in serial coronal slices oriented according to the rat brain atlas of Paxinos and Watson (1998). The location of the probes was reconstructed and referred to the rat brain atlas of Paxinos and Watson (1998) (Figure 1). One rat was excluded from the study for cannula misplacement.

#### Drugs and Treatments

For electrophysiological experiments, morphine hydrochloride (Franchini Prodotti Chimici Srl, Mozzate, Como, Italy) was dissolved in standard aCSF and bath-applied to brain slices at the concentration of 10 μM; ethanol (Sigma-Aldrich, Milan, Italy) was diluted in standard aCSF and bath-applied at the concentration of 80 mM; *WSE* (the standardized dry methanolic extract of *Withania somnifera* roots (Natural Remedies Pvt. Ltd., Bangalore, India) whose certificate of analysis reports that solvent residues were in compliance with the limits envisioned in the British and US Pharmacopoeias) was dissolved in standard aCSF and bath-applied to brain slices at concentrations of 40, 100, 200, and 400 μg/ml. *WSE*, in the absence or presence of either morphine or ethanol, was bath applied in accordance with the following protocol: (i) recording of basal firing activity (baseline) for at least 3–5 min; (ii) perfusion of *WSE* for 5–15 min; (iii) wash-out of variable length (usually between 20 and 40 min, until firing returned to basal control levels); (iv) co-perfusion of *WSE* and morphine or ethanol for 5–15 min. In this respect, both morphine and ethanol were bath-applied twice: the first time (followed by an appropriate wash-out) after a stable baseline was recorded and, the second time, 10 min after the beginning of WSE perfusion. A new baseline was set after WSE induced a stable inhibitory effect on firing rate, and this was used to calculate the action of both drugs.

For *in vivo* microdialysis experiments, morphine hydrochloride (Franchini Prodotti Chimici Srl, Mozzate, Como, Italy) was dissolved in physiological saline [0.9% (w/v) NaCl] and administered subcutaneously (s.c.) at the dose of 5 mg/kg/1 ml; ethanol (Sigma-Aldrich, Milan, Italy), diluted at the concentration of 20% (v/v) in tap water, was administered intragastrically (i.g.) at the dose of 1 g/kg/10 ml. *WSE* (Natural Remedies Pvt. Ltd., Bangalore, India) was dissolved in saline and administered intraperitoneally (i.p.) at the doses, 75 or 100 (data not shown) mg/kg, selected on the basis of our previous studies (Kasture et al., 2009; Ruiu et al., 2013; Peana et al., 2014; Spina et al., 2015). Randomization of animals was made before starting the *in vivo* experiments. Animals were randomized in two different experimental groups, treated with saline or *WSE.*

*WSE* was administered 30 min before morphine treatment, as reported by Ruiu et al. (2013) and Orrù et al. (2014) and 60 min before ethanol treatment, as reported by Spina et al. (2015). Moreover, in order to have *WSE* administered 60 min before (hence, to adopt the same experimental conditions for ethanol and morphine data), we also performed a set of experiments administering *WSE* 60 min before the morphine treatment.

The composition of the standardized methanolic extract of *Withania somnifera* roots (i.e., different batches have similar, and certified, composition with respect to the total withanolides’ content) on the basis of HPLC analysis, as certified by Natural Remedies Pvt. Ltd., Bangalore, India, was as follows: Withanoside-IV: 0.49% (w/w); Physagulin D: 0.11% (w/w); 27-Hydroxywithanone: 0.01% (w/w); Withanoside-V: 0.33% (w/w); Withaferin-A: 0.11% (w/w); 12-Deoxywithastramonolide: 0.16% (w/w); Withanolide-A: 0.19% (w/w); Withanone: 0.004% (w/w); and Withanolide-B: 0.03% (w/w) (Kasture et al., 2009).

#### Statistics

For the electrophysiological experiments, statistical analysis was performed with one-way analysis of variance (ANOVA), followed by Bonferroni’s *post hoc* test with statistical significance set at *P* < 0.05 using Prism 6.0 software (GraphPad Software, La Jolla, CA, United States). The statistical analysis for the microdialysis experiments was carried out by Statistica 8.0 (StatsSoft Inc., Tulsa, OK, United States) for Windows. Basal dialysate DA was calculated as the mean of the last three consecutive samples differing by no more than 10%, collected during the time period preceding each treatment. Basal dialysate DA values, expressed as fmol/10 μl of dialysate, were compared between groups by one-way ANOVAs. Changes in dialysate DA were expressed as percent of the respective baseline values and were analyzed by two-way ANOVAs with repeated measures over time. Results from treatments showing significant overall changes were subjected to *post hoc* Tukey’s test with statistical significance set at *P* < 0.05. Sample size was defined as “n” and clearly indicated for every graph in figures. Due to the type of experiments conducted no blinding approach was made. The number of animal to be used for the experiments was obtained “*a priori*” through GPower software.

## Results

### Electrophysiological Experiments

#### Effect of *WSE* on Spontaneous and Morphine- and Ethanol-Induced Increase in Firing Rate of VTA DA Neurons

**Figure 2:**
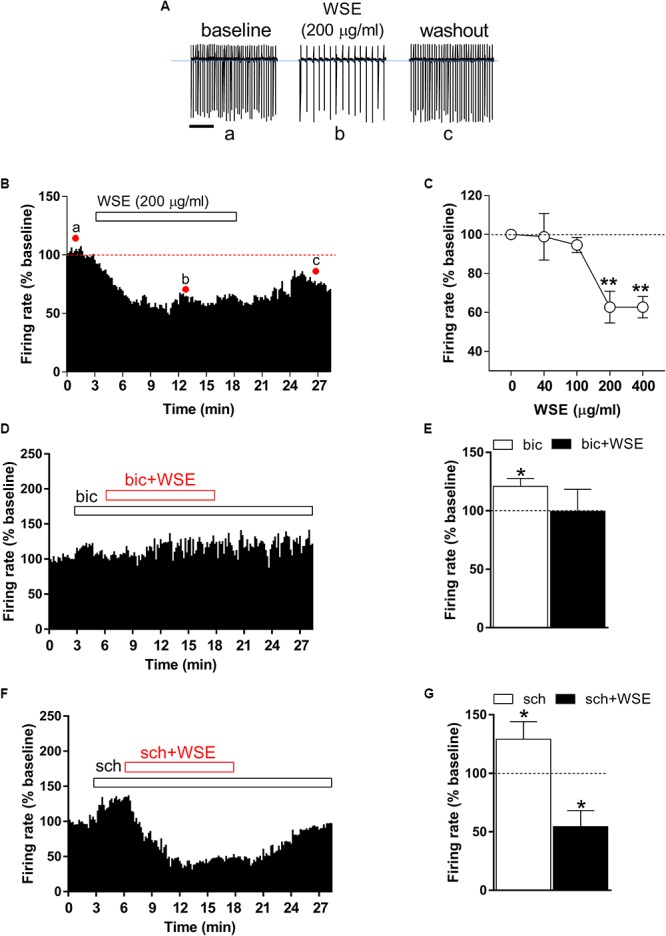
Effect of *WSE* on VTA DA neurons firing rate. **(A)** Representative traces of spontaneous firing recorded in the cell-attached configuration from a single DA neuron before (a: baseline), during (b) and after (c: wash-out) bath-application of *WSE* (200 μg/ml). Scale bar: 1 s. **(B)** The graph shows the temporal changes of firing rate of a single VTA DA neuron before (a: baseline), during (b) and after (c: wash-out) bath-application of *WSE* (200 μg/ml). Values are expressed as percent change from baseline. Points and letters indicate the correspondent section of the trace that was isolated from the whole recording and reported in panel **(A)**. **(C)** The graph reports the concentration-dependent effect of *WSE* on firing rate recorded in VTA DA neurons. Data are expressed as percent change from baseline and are means ± SEM (*n* = 5–32 cells from 10 animals). ^∗∗^*P* < 0.01 vs. baseline. **(D)** The graph shows the temporal changes of firing rate of a single VTA DA neuron during and after (wash-out) bath-application of *WSE* (200 μg/ml) in the presence of the GABA_A_ antagonist bicuculline (bic) 20 μM. Values are expressed as percent change from baseline. **(E)** Bar graph reports the average of different recorded cells (*n* = 5 cells from three animals) in which the effect of bicuculline and co-perfusion of bicuculline and WSE was evaluated. Data are expressed as percent change from baseline and are means ± SEM. ^∗^*P* < 0.05 vs. baseline. **(F)** The histogram graph shows the temporal changes of firing rate of a single VTA DA neuron during and after (wash-out) bath-application of *WSE* (200 μg/ml) in the presence of the GABA_B_ antagonist SCH 50911 10 μM. Values are expressed as percent change from baseline. **(G)** The histogram graph reports the average of different recorded cells (*n* = 5 cells from three animals) in which the effect of SCH 50911(sch) and co-perfusion of SCH 50911 and WSE was evaluated. Data are expressed as percent change from baseline and are means ± SEM. ^∗^*P* < 0.05 vs. baseline.

Identification of single VTA DA neurons was confirmed by the presence of HCN-mediated *I*_h_ currents, evoked by hyperpolarizing the cell membrane from -65 to 115 mV (amplitude, -185 ± 43 pA; *n* = 40) (data not shown), and of a regular spontaneous firing rate (4.84 ± 0.4 Hz, *n* = 40) (Figure 2A, control) (Ungless and Grace, 2012) In addition, the stimulatory effect induced by the GABA_B_ receptor antagonist SCH 50911 (10 μM) (Figure 2G) on firing rate further proved the dopaminergic phenotype of the recorded VTA neurons (Margolis et al., 2012). In order to evaluate quantitatively the effects of *WSE*, alone or in combination with morphine or ethanol, on the spontaneous firing activity of DA neurons in rat VTA slices, spike discharge was recorded in the cell-attached configuration. When applied alone, *WSE* (40–400 μg/ml) significantly decreased the firing rate of VTA DA neurons in a concentration-dependent manner [*F*(4,74) = 10.24, *P* < 0.0001] (Figure 2A–C). A representative effect of *WSE* (200 μg/ml) is depicted in Figure 2A, whereas Figure 2B illustrates the time-course of the action of *WSE* recorded in a single DA neuron. The apparent threshold concentration of *WS*E that was effective in altering DA neuron firing ranged between 100 and 200 μg/ml (Figure 2C). At the highest tested concentrations of 200 or 400 μg/ml, the action of *WSE* resulted in a significant decrease of neuron firing of 37.3 ± 8.1% (*P* < 0.001) and 37.3 ± 5.5% (*P* < 0.001), respectively, compared to baseline.

In order to elucidate the possible mechanism of action through which WSE may mediate its inhibitory effect on firing rate of VTA neurons, we perfused WSE (200 μg/ml) in the presence of GABA_A_ and GABA_B_ receptor selective antagonists, bicuculline (20 μM) and SCH 50911 (10 μM), respectively. As shown in Figure 2D,E, in the presence of bicuculline WSE failed to reduce the firing rate of VTA dopaminergic neurons. It is noteworthy that 3-min perfusion of bicuculline alone elicited a slight but significant increase of the firing rate that may likely depend on the suppression of the GABA_A_-mediated inhibitory input onto DA cells (Figure 2D,E). On the contrary, WSE still decreased the firing rate of VTA neurons in the presence of the GABA_B_ selective antagonist SCH 50911. The perfusion of SCH 50911 alone produced a significant increase of the firing rate (Figure 2F,G), consistent with the selective postsynaptic expression of GABA_B_ receptors on VTA dopaminergic neurons (Margolis et al., 2012).

**Figure 3:**
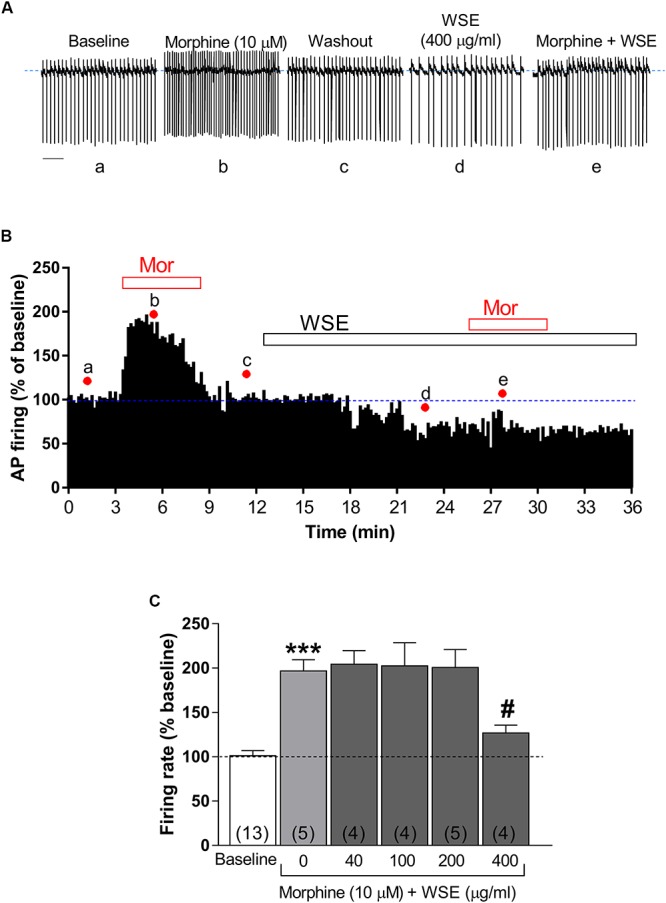
Effects of *WSE* on the stimulatory action of morphine on VTA DA neuron firing rate. **(A)** Representative traces of spontaneous firing recorded form a single DA neuron before (baseline), during and after (wash-out) the bath-application of morphine (10 μM) alone and in the presence of *WSE* (400 μg/ml). Scale bar: 1 s. **(B)** The graph shows the temporal changes of firing rate of a single VTA DA neuron during and after (wash-out) bath-application of morphine (10 μM) (Mor) alone and of morphine in the presence of WSE (400 μg/ml). Values are expressed as percent change from baseline. Points and letters indicate the correspondent section of the trace that was isolated from the whole recording and reported in panel **(A)**. **(C)** The histogram graph shows the concentration–response effect of *WSE* on the stimulatory action of morphine on firing rate. Data are expressed as percent change from baseline and are means ± SEM. The number of cells analyzed (obtained from at least three animals per group of recordings) is indicated by the number inside each histogram. ^∗∗∗^*P* < 0.0001 vs. baseline; ^#^*P* < 0.05 vs. morphine alone.

As expected from previous reports (Gysling and Wang, 1983; Laviolette et al., 2004; Chen et al., 2015; Melis et al., 2015), morphine (10 μM) significantly enhanced the spontaneous firing rate of VTA DA neurons by 96.7 ± 12.8% (*P* < 0.0001 vs. baseline) (Figure 3A–C). Notably, this effect was prevented (*P* < 0.05) in the presence of the highest concentration (400 μg/ml) of *WSE*, whereas lower concentrations resulted completely ineffective [*F*(5,29) = 15.96, *P* < 0.001] (Figure 3C).

**Figure 4:**
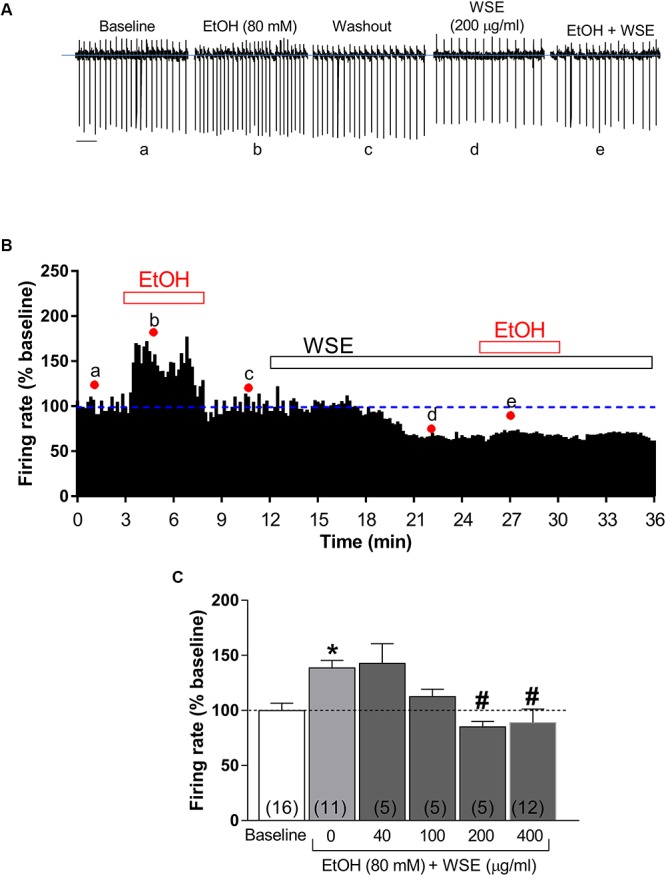
Effects of *WSE* on the stimulatory action of Ethanol on VTA DA neuron firing rate. **(A)** Representative traces of spontaneous firing recorded from a single DA neuron before (baseline), during and after (wash-out) the bath-application of ethanol (80 mM) (EtOH) alone and in the presence of *WSE* (200 μg/ml). Scale bar: 1 s. **(B)** The graph shows the temporal changes of firing rate of a single VTA DA neuron during and after (wash-out) bath-application of EtOH (80 mM) alone and of EtOH in the presence of WSE (200 μg/ml). Values are expressed as percent change from baseline. Points and letters indicate the correspondent section of the trace that was isolated from the whole recording and reported in panel **(A)**. **(C)** The histogram graph shows the concentration–response effect of *WSE* on the stimulatory action of ethanol on firing rate. Data are expressed as percent change from baseline and are means ± SEM. The number of cells analyzed (obtained from at least three animal per group of recordings) is indicated by the number inside each histogram. ^∗^*P* < 0.05 vs. baseline; ^#^*P* < 0.05 vs. ethanol alone.

As previously reported (Brodie et al., 1990; Brodie and Appel, 1998; Xiao and Ye, 2008; Xiao et al., 2009), bath-perfusion with ethanol (80 mM) produced a significant increase of DA neuronal firing rate (38.6 ± 6.8%, *P* < 0.05 vs. baseline) (Figure 4A–C). When each of the different concentrations of *WSE* (40, 100, 200, or 400 μg/ml) was bath-applied for 10 min prior to the co-perfusion with ethanol (80 mM), its stimulatory effect was reduced to 13.2 ± 8.4% by *WSE* at 100 μg/ml, and completely suppressed by *WSE* at 200 and 400 μg/ml (*P* > 0.05) (Figure 4B,C).

### Brain Microdialysis Experiments

Basal AcbSh DA concentration (fmol/sample, mean ± SEM) was on average 55 ± 3 (*n* = 56) and baseline DA did not differ between treatment groups [*F*(12,43) = 0.003; *P* > 0.05].

#### Effect of *WSE* Administration on Morphine- and Ethanol-Elicited Increase of DA Transmission in the AcbSh

**Figure 5:**
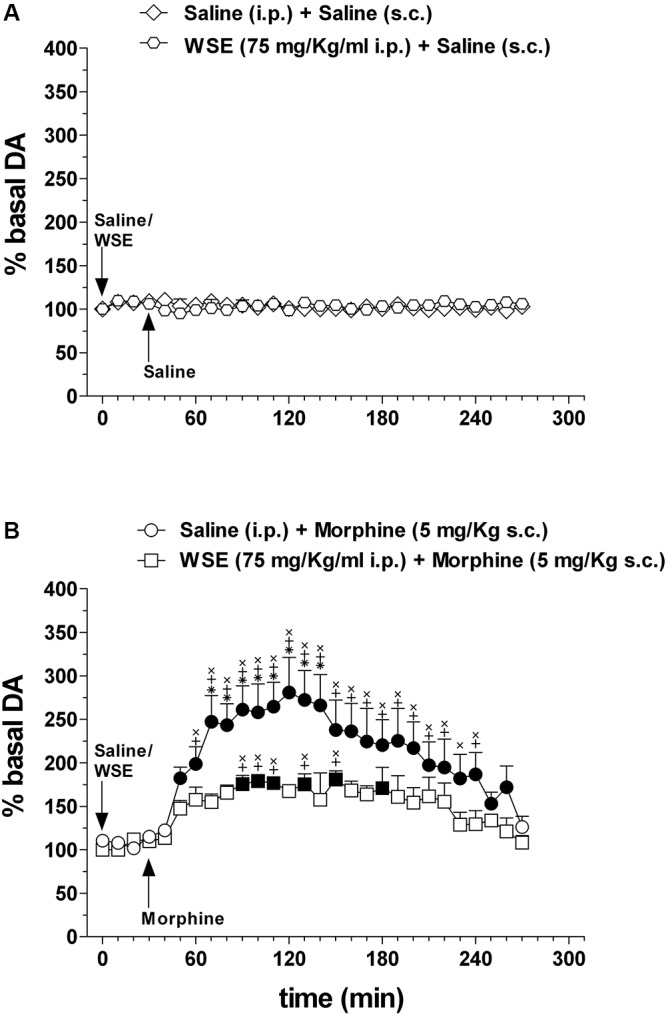
Effects of *WSE* (30 min beforehand) on basal and morphine-elicited stimulation of DA transmission in the AcbSh. **(A)** AcbSh DA responsiveness to saline or *WSE* administration. **(B)** Effect of saline (s.c.) or morphine (5 mg/Kg s.c.) administration on DA transmission in the AcbSh of saline- or *WSE*- pre-treated, 30 min beforehand, rats. Data are means ± SEM of the results, expressed as a percentage of basal values. Filled symbols **(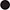/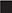)** indicate *P* < 0.05 vs. basal values; ^∗^indicates *P* < 0.05 with respect to *WSE* + morphine; ^+^indicates *P* < 0.05 with respect to *WSE* + saline; ^x^indicates *P* < 0.05 with respect to saline + saline.

Figure 5 shows the effects of saline or *WSE* (75 mg/kg) administration followed, 30 min later, by the administration of saline (Figure 5A) (*N* = 8) or morphine (5 mg/kg) (Figure 5B) (*N* = 8) on AcbSh DA transmission.

Saline or *WSE* administration failed to significantly affect basal DA. Two-way ANOVA of data obtained after saline or *WSE* administration and restricted to the 30 min before saline or morphine administration, revealed a significant effect of time [*F*(3,30) = 16.72; *P* < 0.001] but not of treatment [*F*(3,10) = 0.60, *P* = 0.63] nor a significant treatment × time interaction [*F*(9,30) = 0.39, *P* = 0.93]. However, *post hoc* test failed to reveal any significant DA change after saline or *WSE*, nor difference between groups.

In agreement with previous reports, morphine significantly increased AcbSh DA transmission and *WSE* administration significantly prevented this increase. Two-way ANOVA of data obtained after saline or morphine administration to saline- or *WSE*- pre-treated rats revealed significant effects of time [*F*(24,240) = 8.51, *P* < 0.001] and treatment [*F*(3,10) = 12.87, *P* < 0.01] and a significant treatment × time interaction [*F*(72,240) = 4.07, *P* < 0.001]. *Post hoc* test revealed that morphine significantly increased DA transmission in the AcbSh and that this increase was significantly reduced by *WSE* pre-treatment.

Similarly to the experiments in which *WSE* was administered 30 min before saline or morphine, we found that saline or *WSE* administration failed to significantly affect basal DA. Two-way ANOVA of data obtained after saline or *WSE* administration and restricted to the 60 min before saline or morphine administration, revealed a significant effect of time [*F*(6,84) = 3.67; *P* < 0.01] but not of treatment [*F*(3,14) = 1.42, *P* = 0.28] nor a significant treatment × time interaction [*F*(18,84) = 1.56, *P* = 0.09]. *Post hoc* test failed to reveal significant DA changes after saline or *WSE* and significant differences between groups. Moreover, as in the experiments depicted in Figure 5, morphine (administered 60 min after saline) significantly increased AcbSh DA transmission and WSE administration (60 min before morphine) significantly prevented this increase. Two-way ANOVA of data obtained after saline or morphine administration to saline- or *WSE*- pre-treated rats revealed significant effects of time [*F*(24,336) = 12.28, *P* < 0.01] and treatment [*F*(3,14) = 11.36, *P* < 0.01] and a significant treatment × time interaction [*F*(72,336) = 4.95, *P* < 0.01]. *Post hoc* test revealed that morphine significantly increased DA transmission in the AcbSh and that this increase was significantly prevented by *WSE* pre-treatment (data not shown).

**Figure 6:**
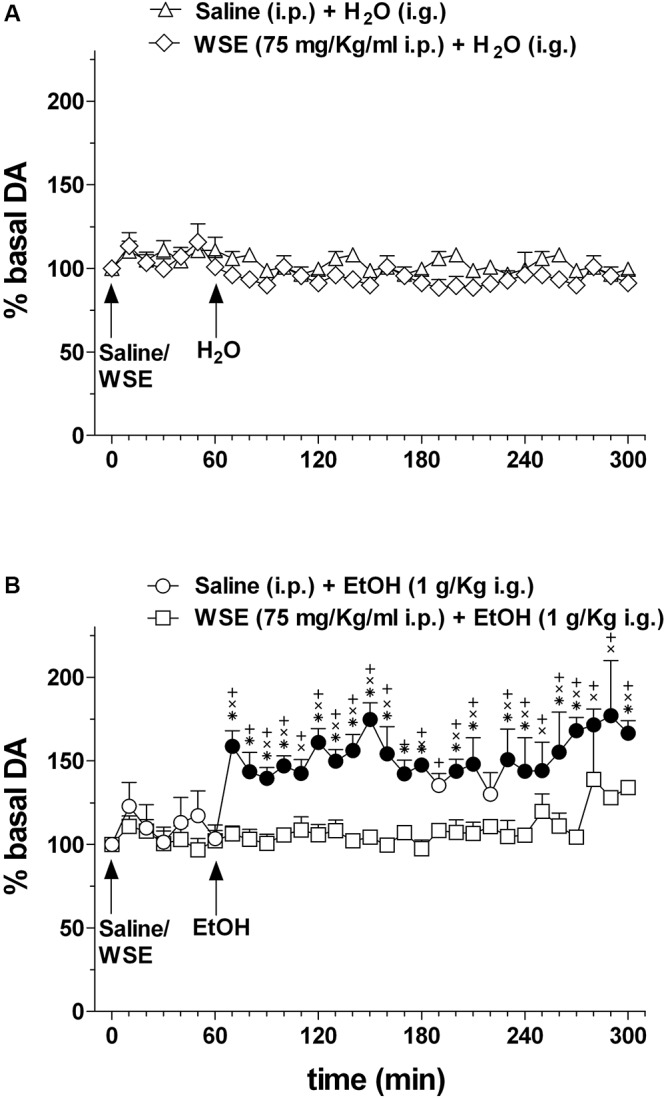
Effects of *WSE* (60 min beforehand) on basal and ethanol-elicited stimulation of DA transmission in the AcbSh. **(A)** AcbSh DA responsiveness to saline or *WSE* administration. **(B)** Effect of tap water (H_2_O) (i.g.) or ethanol (EtOH) (1 g/Kg i.g.) administration on DA transmission in the AcbSh of saline- or *WSE*- pre-treated rats, 60 min beforehand. Data are means ± SEM of the results, expressed as a percentage of basal values. Filled symbols (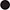/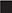) indicate *P* < 0.05 vs. basal values; ^∗^indicates *P* < 0.05 with respect to *WSE* + EtOH; ^+^indicates *P* < 0.05 with respect to *WSE* + H_2_O; ^x^indicates *P* < 0.05 with respect to saline + H_2_O.

Figure 6 shows the effects of saline or *WSE* (75 mg/kg) administration followed, 60 min later, by the administration of water (Figure 6A) (*N* = 8) or ethanol (1 g/kg) (Figure 6B) (*N* = 11) on AcbSh DA transmission.

Saline or *WSE* administration failed to significantly affect basal DA. Two-way ANOVA of data obtained after saline or *WSE* administration and restricted to the 60 min before water or ethanol administration, failed to reveal significant effects of time [*F*(6,84) = 1.95; *P* < 0.08] and treatment [*F*(3,14) = 0.15, *P* = 1.95] and significant treatment × time interaction [*F*(6,84) = 0.63, *P* = 0.86]. Accordingly, *post hoc* test failed to reveal any significant DA change after saline or *WSE* and significant differences between groups.

In agreement with previous reports, ethanol significantly increased AcbSh DA transmission and *WS*E administration prevented this increase. Two-way ANOVA of data obtained after water or ethanol administration to saline- or *WSE*-pre-treated rats revealed significant effects of time [*F*(20,28) = 1.75, *P* < 0.03] and treatment [*F*(3,14) = 23.10, *P* < 0.001] and a significant treatment × time interaction [*F*(20,28) = 1.79, *P* < 0.001]. *Post hoc* test revealed that ethanol significantly increased DA transmission in the AcbSh and that this increase was significantly prevented by *WSE* pre-treatment.

## Discussion

Previous studies demonstrated that the acute administration of the standardized methanolic extract of *WS* roots prevents the acquisition and expression of morphine- and ethanol-elicited motivated behaviors in CPP experiments (Ruiu et al., 2013; Spina et al., 2015) as well as the acquisition and maintenance of oral ethanol self-administration (Peana et al., 2014) but, to date, no experimental evidence has been provided to the mechanistic interpretation of these findings. Therefore, the present study was aimed at evaluating the possibility that such behavioral effects of *WSE* could be based on its ability to affect morphine- and ethanol-stimulated firing activity of VTA DA neurons and DA transmission in the AcbSh. To this end, this study firstly evaluates whether *WSE* would affect the spontaneous firing of VTA DA neurons and the associated basal DA transmission in efferent areas such the AcbSh. The electrophysiological data indicate, for the first time, that bath application of *WSE* to mesencephalic slices of rats, reversibly and concentration-dependently, negatively modulates the spontaneous firing rate of DA neurons in the VTA. In particular, we found that perfusion of VTA slices with WSE resulted in a significant and reversible decrease in firing rate of DA neurons at concentrations of 200 μg/ml and above, an effect that could be blocked in the presence of the GABA_A_ selective receptor antagonist, bicuculline. Secondly, in agreement with previous extensive literature, the study describes that morphine and ethanol consistently increased both the firing rate of DA neurons in the VTA (Gysling and Wang, 1983; Brodie et al., 1990; Brodie and Appel, 1998; Laviolette et al., 2004; Xiao and Ye, 2008; Xiao et al., 2009; Melis et al., 2015) and DA transmission in the AcbSh (Imperato and Di Chiara, 1986; Di Chiara and Imperato, 1988; Bassareo et al., 2003). In particular, as far as the effect of ethanol on firing rate is concerned, we acknowledge that the concentration used (80 mM) could be considered fairly high but, as previously discussed by us (Melis et al., 2015), it may be misleading to make direct comparisons between concentrations (between 20 and 40 mM) attainable upon systemic administration of doses in the range between 1 and 2 g/kg, those achieved upon intra VTA self-administration (between 17 and 66 mM) (Rodd et al., 2005) and, finally, those used in *ex vivo* preparations (in the range between 80 and 100 mM) (Melis et al., 2015 and present data). In addition, in previous studies in which the effects of ethanol on VTA DA neuron firing rate were analyzed (see Brodie et al., 1990; Brodie and Appel, 1998) concentrations of ethanol ranging from 40 to 160 mM were used. The concentration of 80 mM was chosen as it provides a reliable and reproducible effect in our experimental conditions.

Thirdly, the main findings of the present study disclose that *WSE* significantly attenuates the ability of both morphine and ethanol to stimulate spontaneous firing of DA neurons in the VTA and DA transmission in the AcbSh.

The pattern of firing activity of mesencephalic DA neurons varies between low-frequency “tonic” firing activity and brief higher frequency “phasic” bursts of action potentials (Grace and Bunney, 1984), but see for review Rice et al. (2011), and spontaneous firing of DA neurons in the VTA and DA release and transmission in the AcbSh are correlated. However, while the transition from “tonic” to “phasic” activity of DA neurons is correlated with a discrete and marked increase in DA release and DA signaling in the AcbSh (Grace, 1995; Sombers et al., 2009), alterations (decrease or increase) in low-frequency tonic firing activity are not always tightly correlated with parallel changes in AcbSh DA signaling (Hollerman et al., 1992; Grace, 1995). A number of modulatory systems and neurotransmitters in the AcbSh contribute to regulate DA release, including glutamate, GABA, and acetylcholine (Rice et al., 2011). Thus, based on these observations, our data showing that *WSE*, at the concentration of 200 μg/ml and above, reduced basal low-frequency firing activity of VTA DA neurons but did not alter basal DA transmission in the AcbSh, neither at the dose of 75 mg/kg (Figure 5, 6) nor at 100 mg/ml (data not shown), should not be surprising. In addition, we should also consider the obvious difference in the two experimental models used which may imply circuit effects in the intact organism that are not present in the *ex vivo* preparation. In addition, we might also hypothesize that some WSE components that contribute to the effect detected in the *ex vivo* experimental setting may, upon systemic administration, not cross the blood–brain barrier (BBB). Moreover, as it has been shown in other cases in which the effects of a given compound (for instance salsolinol) on DA transmission in the AcbSh were opposite depending on the site of its application [VTA: increase (Deehan et al., 2013); AcbSh: decrease (Hipólito et al., 2009)], it is also plausible that, after its systemic administration, *WSE* may have reached a non-pharmacologically significant concentration within the AcbSh (or virtually any other related area) to exert a local (intra-AcbSh?) action responsible of the lack of effect herewith highlighted by microdialysis experiments. Thus, although at this stage we cannot offer any conclusive demonstration of the mechanism behind this observation, we suggest that both factors, *ex vivo* vs. intact experimental models and crossing vs. not crossing the BBB, may be relevant for its most plausible interpretation. Local microinjection of WSE into discrete brain areas may help to address this issue in a further study.

The observation that *WSE*, bath co-perfused with morphine or ethanol, could significantly prevent their ability to stimulate the spontaneous firing in VTA slices at different concentrations (in the case of morphine, 400 μg/ml, that is two to four times higher than those required on ethanol-elicited neuronal firing), indicates that the mechanism by which *WSE* affects this property of morphine is less sensitive than that at the basis of the ability of ethanol to stimulate DA neuronal firing. In this regard it is critical to point out that the perfusion with morphine or ethanol was accomplished in the presence of WSE and therefore in the condition of inhibition of spontaneous firing. In particular, co-perfusion of *WSE* with morphine or ethanol was performed at the end of a wash-out period that was required to allow morphine- or ethanol-elicited increase of spontaneous firing to recover back to basal (control) levels (as shown in Figure 3B, 4B). Therefore, the effects of *WSE* and those of morphine or ethanol (Brodie et al., 1990; Brodie and Appel, 1998; Xiao and Ye, 2008; Xiao et al., 2009; Chen et al., 2015) should be considered as functionally antagonistic on the stimulation of neuronal firing in the VTA.

In the present study, we also performed specific experiments in order to address the potential molecular mechanism(s) underlying this antagonism. Our results showing that the inhibitory effect of WSE on spontaneous firing of VTA DA neurons was completely abolished by the GABA_A_, but not GABA_B_, receptor selective antagonist bicuculline, suggest that GABA_A_ receptors may be directly involved in this action of WSE on VTA DA neurons. This finding appears in agreement with other reports showing that GABA_A_ receptors are sensitive targets of WSE in different brain regions (Bhattarai et al., 2010; Yin et al., 2013; Candelario et al., 2015). Such results also appear in line with previous receptor binding studies demonstrating that *WSE* shows high to moderate affinities, *k*_i_ values being 13 and 130 μg/ml for GABA_A_ and GABA_B_ receptors, respectively (Ruiu et al., 2013; Orrù et al., 2014). Thus, on the basis of our previous receptor binding studies (Ruiu et al., 2013; Orrù et al., 2014), at the present stage of our investigations on the ability of *WSE* to affect the motivational properties of addictive drugs (Ruiu et al., 2013; Orrù et al., 2014; Spina et al., 2015) we can only hypothesize the involvement of GABA_A_ but not GABA_B_ receptor subtypes in the VTA. Moreover, the possibility that *WSE* might prevent the effects of morphine and ethanol on spontaneous firing through the involvement of DA and opioid receptors should be discarded due to the low affinities of the whole *WS* roots extract for these receptors (Ruiu et al., 2013; Orrù et al., 2014). In further support of the involvement of GABA_A_ receptors in the pharmacological effects of WSE it was shown that WSE activates GABA_A_ receptors-mediated Cl^-^ currents (Bhattarai et al., 2010; Yin et al., 2013; Candelario et al., 2015) and that ashwagandha (*Withania somnifera*) (50–200 mg/kg p.o.) makes animals less seizure-prone by exhibiting dose-dependent anticonvulsant effects [i.e., enhances pentylenetetrazol (PTZ)-induced seizure threshold] in mice (Akula et al., 2009), an interpretation further strengthened by our recent identification into WSE of docosanyl ferulate as a compound able to enhance GABA_A_ inhibitory postsynaptic currents with an IC_50_ of 7.9 μM (Sonar et al., 2019). Altogether, these results encourage to suggest that WSE may antagonize the increase in VTA firing rate induced by both morphine and ethanol by facilitating the function of GABA_A_, but not GABA_B_, receptors located post-synaptically onto the dopaminergic neurons.

On the other hand, our previous work found evidence that the systemic administration of the GABA_B_ receptor antagonist phaclofen prevented the ability of *WSE* to impair maintenance of oral ethanol self-administration (Peana et al., 2014) suggesting also an involvement of GABA_B_ receptors in these *WSE* effects. We can speculate that such GABA_B_ receptor-mediated action of WSE might occur not in the VTA but possibly in the AcbSh where these receptors are expressed in DA fibers (Charara et al., 2000) and whereby their activation decreases DA release (Schmitz et al., 2002; Pitman et al., 2014).

In summary, we speculate that the increased VTA DA neuron firing rate produced in response to both morphine and ethanol may involve a decreased inhibitory GABAergic tone in these cells and that morphine is more effective than ethanol, at least at the concentrations used in the present study. Hence, considering that the antagonism of WSE toward the effect of both drugs may be related with its possible potentiation of the GABAergic system, the different inhibition curve of WSE on the effect of morphine might be explained by the higher stimulation on the firing rate by morphine, and higher inhibition of GABAergic signaling by WSE.

Intriguingly, although not addressed in the present study, at least as far as the effects of WSE on morphine are concerned, another plausible interpretation of the ability of *WSE* to obstruct (functionally antagonize) the stimulatory properties of morphine on DA VTA neurons and DA transmission in the AcbSh, may originate from the observation that *WSE*, similarly to the peroxisome proliferator-activated receptors γ (PPARγ) agonist, pioglitazone (Caputi et al., 2018), has recently been reported to reduce heroin-induced excitation of VTA DA neurons by reducing presynaptic GABA release from the rostromedial tegmental nucleus (De Guglielmo et al., 2015) through the inhibition of nuclear factor-k B (NF-kB).

In our previous studies, we found that the administration of *WSE* could prevent both the acquisition and the expression of morphine- (Ruiu et al., 2013) and ethanol- (Spina et al., 2015) elicited CPP. This is particularly intriguing since the ability of *WSE* to interfere with both phases of the place conditioning paradigm allows to speculate on a further psychopharmacological aspect of the ability of *WSE* to affect morphine’s and ethanol’s motivational properties. In fact, the ability of preventing the acquisition of morphine- and ethanol-elicited CPP (Ruiu et al., 2013; Spina et al., 2015), but also that of ethanol-elicited conditioned place aversion (CPA) (Spina et al., 2015), points to *WSE* as an agent capable of interfering with the direct pharmacological action of morphine and ethanol, i.e., to interfere with the activated biochemical pathway(s) responsible of the behavioral outcome. In other words, *WSE*’s ability to prevent acquisition of drug-elicited place conditioning (either CPP and CPA) seems to suggest its ability to prevent such distinct behavioral outcomes by acting in close proximity of a common mechanism at the basis of the stimulus-stimulus association. In fact, the ability of stimulating DA transmission preferentially (and repeatedly) in the AcbSh, after non-contingent (passive) and contingent exposure (Pontieri et al., 1995, 1996; Tanda et al., 1997; Bassareo et al., 2003; Lecca et al., 2006, 2007a,b; Acquas et al., 2007; Aragona et al., 2008; Bassareo et al., 2017) is common to all drugs of abuse, independently from their mechanism of action. Notably, the continuous stimulation of DA transmission in the AcbSh by drugs of abuse produces an abnormal strengthening of drug–stimulus associations leading to an attribution of excessive motivational value to discrete conditioned stimuli or contexts that become predictive of drug availability. This abnormal association has been hypothesized to be one of the mechanisms that underlies the development of drug addiction (Di Chiara, 1998, 1999; Di Chiara et al., 1999). Hence, by blocking or reducing the increase of AcbSh DA transmission elicited by morphine or ethanol *WSE* might be interfering with their motivational properties and with their ability to produce such abnormal associative learning.

On the other hand, the observation that *WSE* also consistently prevents the expression, in animals in a drug-free state, of the acquired drug-elicited place conditioning (either preference and aversion) calls for a further possible mechanism of action of *WSE* in order to interpret its ability to interfere with the effects of conditioned stimuli on behavior. In this regard, we envision that *WSE* may also interfere with the mechanism at the basis of the ability of conditioned stimuli to elicit increased AcbSh DA transmission (Acquas and Di Chiara, 1994; Di Chiara, 1998; Di Chiara et al., 1999; Bassareo et al., 2007; Bassareo et al., 2011) although we also acknowledge that specific experiments will be required to address the possibility that *WSE*’s ability to prevent also the expression of drug-elicited CPP and CPA encompasses its interference with mesolimbic DA function (Acquas and Di Chiara, 1994; Di Chiara et al., 2004).

## Conclusion

In conclusion, the present results shed fresh light on the neurochemical and receptor-mediated mechanism(s) at the basis of the ability of *WSE* to significantly impact on morphine- and ethanol-elicited motivated behaviors and further support the suggestion of a potential therapeutic application of this extract in drug-contingently and non-contingently motivated behavioral alterations.

## Contribution to the Field Statement

The abuse of addictive substances is a worldwide problem that carries a huge burden to public health. A common characteristic of the central effects of addictive drugs resides in their ability to stimulate the mesolimbic dopaminergic pathway responsible of motivated behaviors. Understanding the mechanisms through which addictive drugs may act in the brain, hijacking the physiological processes that regulate motivated behaviors still represents one of the main targets in the neuroscience field. Accordingly, also the discovery of agents able to antagonize, at different levels, such hijacking processes is a very challenging target. In line with the trend of the last decades by which some natural compounds have proven their efficacy in affecting the central effects of addictive drugs, the findings of the present study reveal that a standardized extract of *Withania somnifera* (Indian ginseng), is able to prevent the ability of morphine and ethanol to increase the activity of dopaminergic neurons by significantly preventing the stimulation neuronal activity and of neurotransmitter release in terminal regions of mesolimbic pathway. Thus, together with previous findings showing that *Withania somnifera* blocks morphine- and ethanol-elicited motivated behaviors, the present results provide a mechanism of these behavioral actions and further point to this extract as a powerful tool to prevent the acute effects of morphine and ethanol.

## Data Availability

The datasets generated for this study are available on request to the corresponding author.

## Ethics Statement

Animal care and handling throughout the experimental procedures were in accordance with the guidelines for care and use of experimental animals of the European Community Council (2010/63/UE L 276 20/10/2010) and with Italian law (DL 04.03.2014, N° 26). In particular, this study was approved by the Organization for Animal Care of the University of Cagliari (OPBA-UniCA) and performed in accordance with the Ministry of Health authorization number 1177/2016-pr (December 15, 2016). Every effort was made to minimize suffering and reduce the number of animals used.

## Author Contributions

EA conceived the study. VB, GT, ES, and EA conceived and designed the experiments. VB, GT, RF, SP, MR, and EL performed the experiments. VB and GT analyzed the data. VB, GT, ES, SK, AP, and EA wrote the manuscript. All authors contributed and approved the final manuscript.

## Conflict of Interest Statement

The authors declare that the research was conducted in the absence of any commercial or financial relationships that could be construed as a potential conflict of interest.

## References

[B1] Acquas E. Di Chiara G. (1994). D1 receptor blockade stereospecifically impairs the acquisition of drug-conditioned place preference and place aversion. *Behav. Pharmacol.* 5 555–569. 10.1097/00008877-199410000-00001 11224235

[B2] Acquas E. Pisanu A. Spiga S. Plumitallo A. Zernig G. Di Chiara G. (2007). Differential effects of intravenous R,S-(+/-)-3,4-methylenedioxy methamphetamine (MDMA, Ecstasy) and its S(+)- and R(-)-enantiomers on dopamine transmission and extracellular signal regulated kinase phosphorylation (pERK) in the rat nucleus accumbens shell and core. *J. Neurochem.* 102 121–132. 10.1111/j.1471-4159.2007.04451.x 17564678

[B3] Akula K. K. Dhir A. Kulkarni S. K. (2009). Effect of various antiepileptic drugs in a pentylenetetrazol-induced seizure model in mice. *Methods Find Exper. Clin. Pharmacol.* 31 423–432. 10.1358/mf.2009.31.7.1393610 19907717

[B4] Aragona B. J. Cleaveland N. A. Stuber G. D. Day J. J. Carelli R. M. Wightman R. M. (2008). Preferential enhancement of dopamine transmission within the nucleus accumbens shell by cocaine is attributable to a direct increase in phasic dopamine release events. *J. Neurosci.* 28 8821–8831. 10.1523/JNEUROSCI.2225-08.2008 18753384 PMC2584805

[B5] Bassareo V. Cucca F. Frau R. Di Chiara G. (2015). Monitoring dopamine transmission in the rat nucleus accumbens shell and core during acquisition of nose-poking for sucrose. *Behav. Brain Res.* 287 200–206. 10.1016/j.bbr.2015.03.056 25827930

[B6] Bassareo V. Cucca F. Frau R. Di Chiara G. (2017). Changes in dopamine transmission in the nucleus accumbens shell and core during ethanol and sucrose self-administration. *Front. Behav. Neurosci.* 11:71. 10.3389/fnbeh.2017.00071 28507512 PMC5410575

[B7] Bassareo V. De Luca M. A. Aresu M. (2003). Differential adaptive properties of accumbens shell dopamine responses to ethanol as a drug and as a motivational stimulus. *Eur. J. Neurosci.* 17 1465–1472. 10.1046/j.1460-9568.2003.02556.x 12713649

[B8] Bassareo V. De Luca M. A. Di Chiara G. (2007). Differential impact of pavlovian drug conditioned stimuli on in vivo dopamine transmission in the rat accumbens shell and core and in the prefrontal cortex. *Psychopharmacology* 191 689–703. 10.1007/s00213-006-0560-7 17072592

[B9] Bassareo V. Musio P. Di Chiara G. (2011). Reciprocal responsiveness of nucleus accumbens shell and core dopamine to food- and drug-conditioned stimuli. *Psychopharmacology* 214 687–697. 10.1007/s00213-010-2072-8 21110007

[B10] Bhatnagar M. Sharma D. Salvi M. (1975). Neuroprotective effects of *Withania somnifera* dunal: a possible mechanism. *Neurochem. Res.* 34 1975–1983. 10.1007/s11064-009-9987-7 19444606

[B11] Bhattacharya A. Ghosal S. Bhattacharya S. K. (2001). Anti-oxidant effect of *Withania somnifera* glycowithanolides in chronic footshock stress-induced perturbations of oxidative free radical scavenging enzymes and lipid peroxidation in rat frontal cortex and striatum. *J. Ethnopharmacol.* 74 1–6. 10.1016/s0378-8741(00)00309-3 11137343

[B12] Bhattarai J. P. Ah Park S. Han S. K. (2010). The methanolic extract of *Withania somnifera* acts on GABAA receptors in gonadotropin releasing hormone (GnRH) neurons in mice. *Phytother. Res.* 24 1147–1150. 10.1002/ptr.3088 20044800

[B13] Brodie M. S. Appel S. B. (1998). The effects of ethanol on dopaminergic neurons of the ventral tegmental area studied with intracellular recording in brain slices. *Alcohol. Clin. Exp. Res.* 22 236–244. 10.1111/j.1530-0277.1998.tb03644.x 9514313

[B14] Brodie M. S. Shefnerand S. A. Dunwiddie T. V. (1990). Ethanol increases the firing rate of dopamine neurons of the rat ventral tegmental area in vitro. *Brain Res.* 508 65–69. 10.1016/0006-8993(90)91118-z 2337793

[B15] Candelario M. Cuellar E. Reyes-Ruiz J. M. Darabedian N. Feimeng Z. Miledi R. (2015). Direct evidence for GABAergic activity of *Withania somnifera* on mammalian ionotropic GABA A and GABA ρ receptors. *J. Ethnopharmacol.* 2 264–272. 10.1016/j.jep.2015.05.058 26068424

[B16] Caputi F. F. Rullo L. Acquas E. Ciccocioppo R. Candeletti S. Romualdi P. (2018). Evidence of a PPAR-mediated mechanism in the ability of *Withania somnifera* to attenuate tolerance to the antinociceptive effects of morphine. *Pharmacol. Res.* 139 422–430. 10.1016/j.phrs.2018.11.033 30503841

[B17] Carai M. A. Agabio R. Bombardelli E. Bourov I. Gessa G. L. Lobina C. (2000). Potential use of medicinal plants in the treatment of alcoholism. *Fitoterapia* 71 S38–S42. 10930711 10.1016/s0367-326x(00)00178-7

[B18] Charara A. Heilman T. C. Levey A. I. Smith Y. (2000). Pre- and postsynaptic localization of GABA(B) receptors in the basal ganglia in monkeys. *Neuroscience* 95 127–140. 10.1016/s0306-4522(99)00409-1 10619469

[B19] Chen M. Zhao Y. Yang H. Luan W. Song J. Cui D. (2015). Morphine disinhibits glutamatergic input to VTA dopamine neurons and promotes dopamine neuron excitation. *eLife* 4:e09275. 10.7554/eLife.09275 26208338 PMC4538365

[B20] Dar N. J. Hamid A. Ahmad M. (2015). Pharmacologic overview of *Withania somnifera*, the Indian Ginseng. *Cell. Mol. Life Sci.* 72 4445–4460. 10.1007/s00018-015-2012-1 26306935 PMC11113996

[B21] Dar P. A. Singh L. R. Kamal M. A. Dar T. A. (2016). Unique Medicinal Properties of *Withania somnifera*: phytochemical constituents and protein component. *Curr. Pharm. Design* 22 535–540. 10.2174/1381612822666151125001751 26601969

[B22] Davis L. Kuttan G. (2001). Effect of *Withania somnifera* on DMBA induced carcinogenesis. *J. Ethnopharmacol.* 75 165–168. 10.1016/s0378-8741(00)00404-9 11297845

[B23] Dazzi L. Talani G. Biggio F. Utzeri C. Lallai V. Licheri V. (2014). Involvement of the cannabinoid CB1 receptor in modulation of dopamine output in the prefrontal cortex associated with food restriction in rats. *PLoS One* 9:e92224. 10.1371/journal.pone.0092224 24632810 PMC3954872

[B24] De Guglielmo G. Melis M. De Luca M. A. Kallupi M. Li H. W. Niswender K. (2015). PPARγ activation attenuates opioid consumption and modulates mesolimbic dopamine transmission. *Neuropsychopharmacology* 40 927–937. 10.1038/npp.2014.268 25311134 PMC4330506

[B25] Deehan G. A. Engleman E. A. Ding Z. M. Engleman E. A. Ding Z. M. McBride W. J. (2013). Microinjections of acetaldehyde or salsolinol into the posterior ventral tegmental area increase dopamine release in the nucleus accumbens shell. *Neuroscience* 37 722–729. 10.1111/acer.12034 23278868 PMC4265471

[B26] Di Chiara G. (1998). A motivational learning hypothesis of the role of mesolimbic dopamine in compulsive drug use. *J. Psychopharmacol.* 12 54–67. 10.1177/026988119801200108 9584969

[B27] Di Chiara G. (1999). Drug addiction as dopamine-dependent associative learning disorder. *Eur. J. Pharmacol.* 375 13–30. 10.1016/s0014-2999(99)00372-6 10443561

[B28] Di Chiara G. Bassareo V. Fenu S. De Luca M. A. Spina L. Cadoni C. (2004). Dopamine and drug addiction: the nucleus accumbens shell connection. *Neuropharmacology* 47 227–241. 10.1016/j.neuropharm.2004.06.032 15464140

[B29] Di Chiara G. Imperato A. (1988). Drugs abused by humans preferentially increase synaptic dopamine concentrations in the mesolimbic system of freely moving rats. *Proc. Natl. Acad. Sci. U.S.A.* 85 5274–5278. 10.1073/pnas.85.14.5274 2899326 PMC281732

[B30] Di Chiara G. Tanda G. Bassareo V. (1999). Drug addiction as a disorder of associative learning. role of nucleus accumbens shell/extended amygdala dopamine. *Ann. N.Y. Acad. Sci.* 29 461–485. 10.1111/j.1749-6632.1999.tb09283.x 10415665

[B31] Di Chiara G. Tanda G. Frau R. Carboni E. (1993). On the preferential release of dopamine in the nucleus accumbens by amphetamine: further evidence obtained by vertically implanted concentric dialysis probes. *Psychopharmacology* 112 v398–v402. 7871048 10.1007/BF02244939

[B32] Diana M. Spiga S. Acquas E. (2006). Persistent and reversible morphine withdrawal-induced morphological changes in the nucleus accumbens. *Ann. N.Y. Acad. Sci.* 1074 446–457. 10.1196/annals.1369.045 17105943

[B33] Gessa G. L. Muntoni F. Collu M. Vargiu L. Mereu G. (1985). Low doses of ethanol activate dopaminergic neurons in the ventral tegmental area. *Brain Res.* 348 201–203. 10.1016/0006-8993(85)90381-6 2998561

[B34] Grace A. A. (1995). The tonic/phasic model of dopamine system regulation: its relevance for understanding how stimulant abuse can alter basal ganglia function. *Drug Alcohol Depend.* 37 111–129. 10.1016/0376-8716(94)01066-t 7758401

[B35] Grace A. A. Bunney B. S. (1984). The control of firing pattern in nigral dopamine neurons: burst firing *J. Neurosci.* 4 2877–2890. 10.1523/jneurosci.04-11-02877.1984 PMC6564720 6150071

[B36] Grace A. A. Onn S. P. (1989). Morphology and electrophysiological properties of immunocytochemically identified rat dopamine neurons recorded in vitro. *J. Neurosci.* 9 3463–3481. 10.1523/jneurosci.09-10-03463.1989 2795134 PMC6569889

[B37] Gupta G. L. Rana A. C. (2008). Effect of *Withania somnifera* Dunal in ethanol-induced anxiolysis and withdrawal anxiety in rats. *Indian J. Exp. Biol.* 46 470–475. 18697607

[B38] Gysling K. Wang R. Y. (1983). Morphine-induced activation of A10 dopamine neurons in the rat. *Brain Res.* 277 119–127. 10.1016/0006-8993(83)90913-7 6315137

[B39] Hipólito L. Sánchez-Catalán M. J. Granero L. Polache A. (2009). Local salsolinol modulates dopamine extracellular levels from rat nucleus accumbens: shell/core differences. *Neurochem. Int.* 55 187–192. 10.1016/j.neuint.2009.02.014 19524107

[B40] Hollerman J. R. Abercrombie E. D. Grace A. A. (1992). Electrophysiological, biochemical, and behavioral studies of acute haloperidol-induced depolarization block of nigral dopamine neurons. *Neuroscience* 47 589–601. 10.1016/0306-4522(92)90168-2 1584410

[B41] Imperato A. Di Chiara G. (1986). Preferential stimulation of dopamine release in the nucleus accumbens of freely moving rats by ethanol. *J. Pharmacol. Exp. Ther.* 239 219–228. 3761194

[B42] Jalabert M. Bourdy R. Courtin J. Veinante P. Manzoni O. J. Barrot M. (2011). Neuronal circuits underlying acute morphine action on dopamine neurons. *Proc. Natl. Acad. Sci. U.S.A.* 108 16446–16450. 10.1073/pnas.1105418108 21930931 PMC3182694

[B43] Kasture S. Vinci S. Ibba F. Puddu A. Marongiu M. Murali B. (2009). Withania somnifera prevents morphine withdrawal-induced decrease in spine density in nucleus accumbens shell of rats: a confocal laser scanning microscopy study. *Neurotox. Res.* 16 343–355. 10.1007/s12640-009-9069-2 19551457

[B44] Kulkarni S. K. Akula K. K. Dhir A. (2008). Effect of *Withania somnifera* Dunal root extract against pentylenetetrazol seizure threshold in mice: possible involvement of GABAergic system. *Indian J. Exper. Biol.* 46 465–469. 18697606

[B45] Kulkarni S. K. Dhir A. (2008). Withania somnifera: an Indian ginseng. *Progr. Neuro-Psychopharmacol. Biol. Psych.* 32 1093–1105. 10.1016/j.pnpbp.2007.09.011 17959291

[B46] Kulkarni S. K. Ninan I. (1997). Inhibition of morphine tolerance and dependence by *Withania somnifera* in mice. *J. Ethnopharmacol.* 57 213–217. 10.1016/s0378-8741(97)00064-0 9292416

[B47] Laviolette S. R. Gallegos R. A. Henriksen S. J. van der Kooy D. (2004). Opiate state controls bi-directional reward signaling via GABAA receptors in the ventral tegmental area. *Nat. Neurosci.* 7 160–169. 10.1038/nn1182 14730310

[B48] Lecca D. Cacciapaglia F. Valentini V. Cacciapaglia F. Acquas E. Di Chiara G. (2007a). Differential neurochemical and behavioral adaptation to cocaine after response contingent and noncontingent exposure in the rat. *Psychopharmacology* 191 653–667. 10.1007/s00213-006-0496-y 16932924

[B49] Lecca D. Valentini V. Cacciapaglia F. Acquas E. Di Chiara G. (2007b). Reciprocal effects of response contingent and noncontingent intravenous heroin on in vivo nucleus accumbens shell versus core dopamine in the rat: a repeated sampling microdialysis study. *Psychopharmacology* 194 103–116. 10.1007/s00213-007-0815-y 17541779

[B50] Lecca D. Cacciapaglia F. Valentini V. Gronli J. Spiga S. Di Chiara G. (2006). Preferential increase of extracellular dopamine in the rat nucleus accumbens shell as compared to that in the core during acquisition and maintenance of intravenous nicotine self-administration. *Psychopharmacology* 184 435–446. 10.1007/s00213-005-0280-4 16397746

[B51] Liu Q. Lawrence A. J. Liang J. H. (2011). Traditional Chinese medicine for treatment of alcoholism: from ancient to modern. *Am. J. Chin. Med.* 39 1–13. 10.1142/s0192415x11008609 21213394

[B52] Lu L. Liu Y. Zhu W. (2009). Traditional medicine in the treatment of drug addiction. *Am. J. Drug Alcohol Abuse* 35 1–11. 10.1080/00952990802455469 19152199

[B53] Lyon J. (2017). More treatments on deck for alcohol use disorder. *JAMA* 317 2267–2269. 28538998 10.1001/jama.2017.4760

[B54] Margolis E. B. Toy B. Himmels P. Morales M. Fields H. L. (2012). Identification of rat ventral tegmental area GABAergic Neurons. *PLoS One* 7:e42365. 10.1371/journal.pone.0042365 22860119 PMC3409171

[B55] Mehta A. K. Binkley P. Gandhi S. S. Ticku M. K. (1991). Pharmacological effects of *Withania somnifera* root extract on GABAA receptor complex. *Indian J. Med. Res.* 94 312–315. 1660034

[B56] Melis M. Carboni E. Caboni P. Acquas E. (2015). Key role of salsolinol in ethanol actions on dopamine neuronal activity of the posterior ventral tegmental area. *Addict. Biol.* 20 182–193. 10.1111/adb.12097 24103023

[B57] Orrù A. Marchese G. Casu G. Casu M. A. Kasture S. Cottiglia F. (2014). Withania somnifera root extract prolongs analgesia and suppresses hyperalgesia in mice treated with morphine. *Phytomedicine* 21 745–752. 10.1016/j.phymed.2013.10.021 24268297

[B58] Paxinos G. Watson C. (1998). *The Rat Brain in Stereotaxic Coordinates*, 4th Edn SanDiego, CA: Academic Press.

[B59] Peana A. T. Muggironi G. Spina L. Rosas M. Kasture S. B. Cotti E. (2014). Effects of *Withania somnifera* on oral ethanol self-administration in rats. *Behav. Pharmacol.* 25 618–628. 10.1097/FBP.0000000000000078 25115596

[B60] Pitman K. A. Puil E. Borgland S. L. (2014). GABA_B_ modulation of dopamine release in the nucleus accumbens core. *Eur. J. Neurosci.* 40 3472–3480. 10.1111/ejn.12733 25229321

[B61] Pontieri F. E. Tanda G. Di Chiara G. (1995). Intravenous cocaine, morphine, and amphetamine preferentially increase extracellular dopamine in the “shell” as compared with the “core” of the rat nucleus accumbens. *Proc. Natl. Acad. Sci. U.S.A.* 92 12304–12308. 10.1073/pnas.92.26.12304 8618890 PMC40345

[B62] Pontieri F. E. Tanda G. Orzi F. Di Chiara G. (1996). Effects of nicotine on the nucleus accumbens and similarity to those of addictive drugs. *Nature* 382 255–257. 10.1038/382255a0 8717040

[B63] Prakash J. Chouhan S. Yadav S. K. Westfall S. Rai S. N. Singh S. P. (2014). *Withania somnifera* alleviates parkinsonian phenotypes by inhibiting apoptotic pathways in dopaminergic neurons. *Neurochem. Res.* 39 2527–2536. 10.1007/s11064-014-1443-7 25403619

[B64] Rice M. E. Patel J. C. Cragg S. J. (2011). Dopamine release in the basal ganglia. *Neuroscience* 198 112–137. 10.1016/j.neuroscience.2011.08.066 21939738 PMC3357127

[B65] Rodd Z. A. Bell R. L. Zhang Y. Murphy J. M. Goldstein A. Zaffaroni A. (2005). Regional heterogeneity for the intracranial self-administration of ethanol and acetaldehyde within the ventral tegmental area of alcohol-preferring (P) rats: involvement of dopamine and serotonin. *Neuropsychopharmacology* 30 330–338. 10.1038/sj.npp.1300561 15383830

[B66] Ruiu S. Longoni R. Spina L. Orrù A. Cottiglia F. Collu M. (2013). Withania somnifera prevents acquisition and expression of morphine-elicited conditioned place preference. *Behav. Pharmacol.* 24 133–143. 10.1097/FBP.0b013e32835f3d15 23455447

[B67] Schliebs R. Liebmann A. Bhattacharya S. K. Kumar A. Ghosa S. Bigl V. (1997). Systemic administration of defined extracts from *Withania somnifera* (Indian Ginseng) and Shilajit differentially affects cholinergic but not glutamatergic and GABAergic markers in rat brain. *Neurochem. Int.* 30 181–190. 10.1016/s0197-0186(96)00025-3 9017665

[B68] Schmitz Y. Schmauss C. Sulzer D. (2002). Altered dopamine release and uptake kinetics in mice lacking D_2_ receptors. *J. Neurosci.* 22 8002–8009. 10.1523/JNEUROSCI.22-18-08002.2002 12223553 PMC6758092

[B69] Singh N. Bhalla M. de Jager P. Gilca M. (2011). An overview on ashwagandha: a Rasayana (rejuvenator) of Ayurveda. *Afr. J. Trad. Compl. Alt. Med.* 5(Suppl.), 208–213. 10.4314/ajtcam.v8i5S.9 22754076 PMC3252722

[B70] Sombers L. A. Beyene M. Carelli R. M. Wightman R. M. (2009). Synaptic overflow of dopamine in the nucleus accumbens arises from neuronal activity in the ventral tegmental area. *J. Neurosci.* 29 1735–1742. 10.1523/JNEUROSCI.5562-08.2009 19211880 PMC2673986

[B71] Sonar V. Fois B. Distinto S. Maccioni E. Meleddu R. Cottiglia F. (2019). Ferulic acid esters and withanolides: in search of *Withania somnifera* GABAA receptor modulators. *J. Nat. Prod.* 10.1021/acs.jnatprod.8b01023 [Epub ahead of print]. 30998355

[B72] Spina L. Longoni R. Rosas M. Collu M. Peana A. T. Espa E. (2015). *Withania somnifera* Dunal (Indian ginseng) impairs acquisition and expression of ethanol-elicited conditioned place preference and conditioned place aversion. *J. Psychopharmacol.* 29 1191–1199. 10.1177/0269881115600132 26349555

[B73] Talani G. Biggio G. Sanna E. (2011). Enhanced sensitivity to ethanol-induced inhibition of LTP in CA1 pyramidal neurons of socially isolated C57BL/6J mice: role of neurosteroids. *Front. Endocrinol.* 2:56. 10.3389/fendo.2011.00056 22649377 PMC3355925

[B74] Talani G. Licheri V. Biggio F. Locci V. Mostallino M. C. Secci P. P. (2016). Enhanced glutamatergic synaptic plasticity in the hippocampal ca1 field of food-restricted rats: involvement of CB1 receptors. *Neuropsychopharmacology* 41 1308–1318. 10.1038/npp.2015.280 26354043 PMC4793114

[B75] Tanda G. Bassareo V. Di Chiara G. (1996). Mianserin markedly and selectively increases extracellular dopamine in the prefrontal cortex as compared to the nucleus accumbens of the rat. *Psychopharmacology* 123 127–130. 10.1007/bf02246169 8741935

[B76] Tanda G. Pontieri F. E. Di Chiara G. (1997). Cannabinoid and heroin activation of mesolimbic dopamine transmission by a common mu1 opioid receptor mechanism. *Science* 276 2048–2050. 10.1126/science.276.5321.2048 9197269

[B77] Theile J. W. Morikawa H. Gonzales R. A. Morrisett R. A. (2011). GABAergic transmission modulates ethanol excitation of ventral tegmental area dopamine neurons. *Neuroscience* 172 94–103. 10.1016/j.neuroscience.2010.10.046 20974231 PMC3010434

[B78] Ungless M. A. Grace A. A. (2012). Are you or aren’t you? challenges associated with physiologically identifying dopamine neurons. *TiNS* 35 422–430. 10.1016/j.tins.2012.02.003 22459161 PMC3383926

[B79] Volkow N. D. Morales M. (2015). The brain on drugs: from reward to addiction. *Cell* 162 712–725. 10.1016/j.cell.2015.07.046 26276628

[B80] Xiao C. Shao X. M. Olive M. F. Griffin W. C. III Li K. Y. Krnjević K. (2009). Ethanol facilitates glutamatergic transmission to dopamine neurons in the ventral tegmental area. *Neuropsychopharmacology* 34 307–318. 10.1038/npp.2008.99 18596684 PMC2676579

[B81] Xiao C. Ye J. H. (2008). Ethanol dually modulates GABAergic synaptic transmission onto dopaminergic neurons in ventral tegmental area: role of mu-opioid receptors. *Neuroscience* 153 240–248. 10.1016/j.neuroscience.2008.01.040 18343590 PMC2424267

[B82] Yenisetti S. C. Manjunath M. J. Muralidhara C. (2016). Neuropharmacological properties of *Withania somnifera* - indian ginseng: an overview on experimental evidence with emphasis on clinical trials and patents. *Recent Pat. CNS Drug Discov.* 10 204–215. 27316579 10.2174/1574889810666160615014106

[B83] Yin H. Cho D. H. Park S. J. (2013). GABA-mimetic actions of *Withania somnifera* on substantia gelatinosa neurons of the trigeminal subnucleus caudalis in mice. *Am. J. Chin. Med.* 41 1043–1051. 10.1142/S0192415X13500705 24117067

[B84] Zhang H. Cao C. M. Gallagher R. J. Timmermann B. N. (2014). Antiproliferative withanolides from several solanaceous species. *Nat. Prod. Res.* 28 1941–1951. 10.1080/14786419.2014.919286 24871278 PMC4177291

